# The subway microbiome: seasonal dynamics and direct comparison of air and surface bacterial communities

**DOI:** 10.1186/s40168-019-0772-9

**Published:** 2019-12-19

**Authors:** Jostein Gohli, Kari Oline Bøifot, Line Victoria Moen, Paulina Pastuszek, Gunnar Skogan, Klas I. Udekwu, Marius Dybwad

**Affiliations:** 1 Norwegian Defence Research Establishment, P.O. Box 25, NO-2027 Kjeller, Norway; 2 Department of Analytics, Environmental & Forensic Sciences, King’s College London, 150 Stamford Street, London, SE1 9NH UK; 3 Department of Molecular Biosciences, Stockholm University, SE 10691 Stockholm, Sweden; 4 SoS BIOs Sweden, Tiundagatan 41, SE 752 30 Uppsala, Sweden

**Keywords:** 16S rRNA gene, Aerosol, Air, Amplicon sequencing, Microbiome, Seasonal variation, Subway

## Abstract

**Background:**

Mass transit environments, such as subways, are uniquely important for transmission of microbes among humans and built environments, and for their ability to spread pathogens and impact large numbers of people. In order to gain a deeper understanding of microbiome dynamics in subways, we must identify variables that affect microbial composition and those microorganisms that are unique to specific habitats.

**Methods:**

We performed high-throughput 16S rRNA gene sequencing of air and surface samples from 16 subway stations in Oslo, Norway, across all four seasons. Distinguishing features across seasons and between air and surface were identified using random forest classification analyses, followed by in-depth diversity analyses.

**Results:**

There were significant differences between the air and surface bacterial communities, and across seasons. Highly abundant groups were generally ubiquitous; however, a large number of taxa with low prevalence and abundance were exclusively present in only one sample matrix or one season. Among the highly abundant families and genera, we found that some were uniquely so in air samples. In surface samples, all highly abundant groups were also well represented in air samples. This is congruent with a pattern observed for the entire dataset, namely that air samples had significantly higher within-sample diversity. We also observed a seasonal pattern: diversity was higher during spring and summer. Temperature had a strong effect on diversity in air but not on surface diversity. Among-sample diversity was also significantly associated with air/surface, season, and temperature.

**Conclusions:**

The results presented here provide the first direct comparison of air and surface bacterial microbiomes, and the first assessment of seasonal variation in subways using culture-independent methods. While there were strong similarities between air and surface and across seasons, we found both diversity and the abundances of certain taxa to differ. This constitutes a significant step towards understanding the composition and dynamics of bacterial communities in subways, a highly important environment in our increasingly urbanized and interconnect world.

Video abstract.

## Background

Microorganisms are ubiquitous in the truest sense of the word; regardless of where humans reside, they are subjected to a plethora of microbes, some of which have profound impacts on our health [1, 2], and our individual microbiomes [3]. The built environment (BE) is the primary environment of modern humans [4], and hence where we mainly encounter microorganisms. Mass transit environments such as subways facilitate a constant flow of microbes among humans and among different BEs [5]. They are thus particularly important for human health due to their potential for spreading pathogens [6] and impact large numbers of people.

The subway surface microbiome has already been characterized using both 16S rRNA gene amplicon sequencing [5, 7] and shotgun metagenomics [5, 8, 9]. Notably, the MetaSUB project [10] has produced a microbiome and antimicrobial resistance atlas from mass transit surface samples spanning 58 cities across the world [11]. Subway air—which is of particular interest with regard to bioterrorism [12] and infectious diseases [13]—has also been studied using both culture-based [14–17] and more recently culture-independent approaches [18–21]. Robertson et al. [21] described the composition and diversity of subway air microbiomes in New York. Leung et al. [20] found extensive bacterial diversity in the air of the Hong Kong subway system, showing that changes in microbial communities were governed by temperature, humidity, and the number of commuters. Triadó-Margarit et al. [19] investigated air microbiomes in the Barcelona subway system and found significant overlap among different environments within the subway and dominance of a few widespread groups of microorganisms. Fan et al. [18] observed variation in the fungal and bacterial air microbiomes of the Beijing Subway between peak and non-peak commuting hours. While many studies have addressed subway air or surface microbiomes separately, to our knowledge, no study has yet provided a direct comparison of these two important and probably closely interacting sample matrices.

Seasonality is a time-dependent, fundamental shift in environmental conditions that is expected to vary greatly across geographical scales. It is well known that outdoor microbiomes show significant variation across seasons [22–24] and that outdoor air strongly contributes to indoor microbiome composition [25]. Hence, seasonal effects on BE microbiomes are to be expected. However, two BE studies of seasonal microbiome variation, one in Finnish office buildings [26] and another at a children’s daycare center in Virginia, USA [27], found no significant seasonal trends. Patel et al. [28] cultivated bacteria from dust collected at railway stations in England and Scotland and found seasonal trends in bacterial abundance, and Heo et al. [14] found concentrations of culturable bacteria in underground subway air to vary across seasonal transitions; however, culture-independent methods have not been utilized to evaluate seasonal microbial diversity in subways or similar environments.

In the present study, we analyzed surface and air samples collected at 16 subway stations in Oslo, Norway, a relatively small capital city—compared with cities previously studied in this context—at the northern boundary of the temperate region. The aim of this work is to provide a direct comparison of surface and air bacterial microbiomes—to identify unique and ubiquitous taxa and to quantify differences in diversity among these sample matrices. Furthermore, we address an important knowledge gap, namely that of seasonal dynamics in subway air and surface bacterial microbiomes. The main hypotheses tested here are (1) that bacterial microbiome composition and diversity varies significantly across seasons, and (2) that bacterial microbiomes found on surfaces and in air differ with regard to composition and diversity.

## Methods

Air (69) and surface (177) samples were collected at 16 subway stations in Oslo, Norway across four seasons from November 2016–June 2017 (Additional file 1: Tables S1 and S2). At each sampling location, one air sample and three surface samples were collected. An Aerotrak 8220 (TSI, Shoreview, MN, US) optical particle counter fitted with an external probe (Model: 1300102) was used to record temperature and humidity.

### Air sampling

Air samples were collected and air filters extracted as previously described in Bøifot et al. [29]. Briefly, particulates in air were collected on an electret filter with a SASS3100 air sampler for 30 min, and at 300 L of air per minute (Research International, Monroe, WA, USA). Filters were placed in 50-mL centrifuge tubes and stored in a transport cooler with ice packs before they were transferred to − 80 °C upon return to the laboratory. Particulates were extracted from the filter with NucliSENS lysis buffer (10 mL, BioMérieux, Marcy-l’Étoile, France), and the filter extract was centrifuged at 7000*g* for 30 min. The supernatant was transferred to a new 50-mL tube, while the centrifuged pellet was resuspended with PowerBead Solution (550 μL, Qiagen, Hilden, Germany) and transferred to autoclaved (121 °C, 45 min) bead tubes (2 mL, Sarstedt, Nümbrecht, Germany) filled with 600 mg, 0.1-mm zirconia/silica beads (BioSpec Products, Bartlesville, OK, USA). PowerSoil Solution C1 (60 μL, Qiagen) was added and bead beating (1 min, maximum intensity) was performed in a Mini-Beadbeater-8 (BioSpec Products). Bead tubes were centrifuged at 13,000g for 2 min and potential inhibitors removed according to the DNeasy PowerSoil Kit with Solution C2 (250 μL) and C3 (200 μL). Following inhibitor removal, we combined the supernatant with that from the filter extract and isolated DNA according to the manufacturer’s protocol of the NucliSENS Magnetic Extraction Reagents Kit (BioMérieux). Negative controls (reagents) were prepared by processing blank samples (unexposed filters) along with the air samples.

### Surface sampling

Surface samples were collected for three surface types at each station: kiosks, railings, and benches. A nylon, flocked swab (Copan eSwab 490 CE.A, Copan Diagnostics, CA, USA) wetted in Amies liquid medium, was used to swab the surface for 3 min, covering an area as large as possible. The swab was placed in a 15-mL centrifuge tube and stored in a transport cooler with ice packs before they were transferred to − 80 °C upon return to the laboratory. DNA was isolated according to the DNeasy PowerSoil Kit (Qiagen) protocol, except that the standard PowerBead Tubes were replaced with the customized bead tubes described above for air samples. Swabs were cut with sterilized scissors into bead tubes filled with PowerBead Solution (550 μL, Qiagen) and Solution C1 (60 μL, Qiagen) before tubes were bead beaten (1 min, maximum intensity) in a Mini-Beadbeater-8 (BioSpec Products). Negative controls (reagents) were prepared by cutting clean swabs into bead tubes and performing DNA isolation.

ZymoBIOMICS Microbial Community Standard (10 μL, Zymo Research) was added to one bead tube before DNA isolation (isolated according the protocol described above), which served as a positive control.

### Quantification of total DNA and bacterial 16S rRNA gene copies

DNA yield was measured with Qubit 3.0 Fluorimeter (Life Technologies, Carlsbad, CA, USA) using the Qubit dsDNA HS assay (Life Technologies). Bacterial 16S rRNA gene copy yield was determined with a qPCR assay performed according to Liu et al. [22] on a LightCycler 480 (Roche Diagnostics, Oslo, Norway). A standard curve was generated with serial dilutions of *Escherichia coli* DNA (seven 16S rRNA gene copies per genome). Bacterial 16S rRNA gene copy yields were analyzed with linear models in R [30]. Given that air and surface samples were collected with different sampling protocols, the data was grouped by air and surface prior to analysis. Surface type, surface material, surface treatment, season, indoor/outdoor station, time of day, temperature (mean and standard deviation), humidity (mean and standard deviance), and sequence run were included as predictors in these models, which were subsequently subjected to a stepwise model (predictor variable) selection with the stepAIC R function [31].

### 16S rRNA gene amplicon sequencing

The 16S rRNA gene was amplified by PCR (Additional file 1: Table S3), using forward primer 341F, 5′-CCTACGGGNGGCWGCAG-3′ and reverse primer 805R, 5′-GGACTACHVGGGTWTCTAAT-3′, targeting the V3 and V4 regions of the gene. Sequence libraries were prepared following the 16S Metagenomic Sequencing Library Preparation protocol [32] and sequenced on Illumina MiSeq in four separate runs. Four swab negative controls, three air negative controls, and one ZymoBIOMICS Microbial Community Standard positive control were included as study controls.

### Sequence analysis

Primers and adapters were removed from demultiplexed sequence reads using TrimGalore [33], a perl wrapper for Cutadapt [34] and FastQC [35]. A big data pipeline, i.e., forward reads only, was used to infer amplicon sequence variants (ASV) using the dada2 R package [36]. Filtering was performed with the filterAndTrim function in dada2; reads that mapped to the phiX genome were removed, all reads were truncated to 250 bp, and reads of < 250 bp, that contained any unassigned bases or bases of quality score < 2, were discarded, and the maximum number of expected read errors per read was set to 2. Learned error rates were used for inferring ASV before removing chimeras (dada2 functionality). Dada2 analyses were run separately for the four sequence runs, before merging the feature tables. SILVA SSU v.132 [37], which is the largest dedicated 16S taxonomy database, was used for assigning taxonomy to the ASVs. The ASV table, taxonomy table, and metadata were imported into the phyloseq R package [30] for analyses.

Reads not assigned to the phylum level were removed before rarefication. All samples were rarified to the lowest read depth after assessing rarefication curves with observed diversity and Shannon’s diversity index. The data set was split into air and surface samples, and into surface types, before summarizing the most abundant phyla, families, and genera in both subsets.

Three random forest classification analyses were performed with 10,001 trees, using air/surface, the four seasons, and surface type as classification bins. ASVs not assigned to genus were discarded before conglomerating all ASVs to the level of genus. A prevalence filter of < 10 and a total abundance filter of < 20 were implemented prior to calculating *Z*-scores from abundances for the remaining 817 genera. The most important features (genera) for correctly assigning samples to their correct bin (air/surface, season, or surface type) was identified using mean decrease in model accuracy (MDA), i.e., the negative impact on model accuracy by excluding a feature.

Shannon’s diversity index scores were analyzed using linear models in R [31]. Firstly, variables only relevant for surface samples (surface type, material, and treatment (painted/not painted)) were analyzed. Second, air/surface, season, indoor/outdoor station, time of day, temperature (mean and standard deviance), humidity (mean and standard deviance), and sequence run, along with all possible two-way interactions, were included as predictors of Shannon’s diversity index scores in a separate model. This model was subjected to a stepwise model selection with the stepAIC R function [38].

Prior to analyses of among-sample diversity, ASVs with prevalence < 5 were removed. A Bray–Curtis dissimilarity matrix was ordinated using PCoA, and PERMANOVA tests were performed using the same predictor variables (including all two-way interactions) mentioned above. Manual AIC model selection was performed by dropping the least significant variable in a step-wise fashion, until further removals no longer improved the model’s AIC score.

## Results

All negative controls (four swabs and three air samples) failed to generate sequenceable libraries in the library preparation step due to insufficient DNA yields. The positive controls showed no sign of contamination and yielded the correct genera. Analyses of 16S rRNA gene copy yields found that bacterial numbers decreased with increasing humidity, peaked during spring for air samples (Additional file 1: Table S4; Figure S1), and were highest during summer, at outdoor stations, and on kiosks for surface samples (Additional file 1: Table S4; Figure S2). For surface samples, the number of 16S rRNA gene copies was also significantly higher in one of the sequence runs (Additional file 1: Table S4; Figure S2).

After QC filtering, 41 M forward reads remained (Additional file 1: Figure S3). A total of 12.6% were lost in the ASV inference step (dada2, with error modeling), and a further 15.1% were removed as chimeras, leaving 30.7 M forward reads. From this material, dada2 identified 328,615 ASVs. A total of 13,788 of these were not assigned to phylum and removed. Rarefication curves for observed diversity and Shannon’s diversity index (Additional file 1: Figure S4) were evaluated before rarefying all samples to a common read depth of 6358, which removed only three samples.

### Taxonomy and community composition

**Fig. 1:**
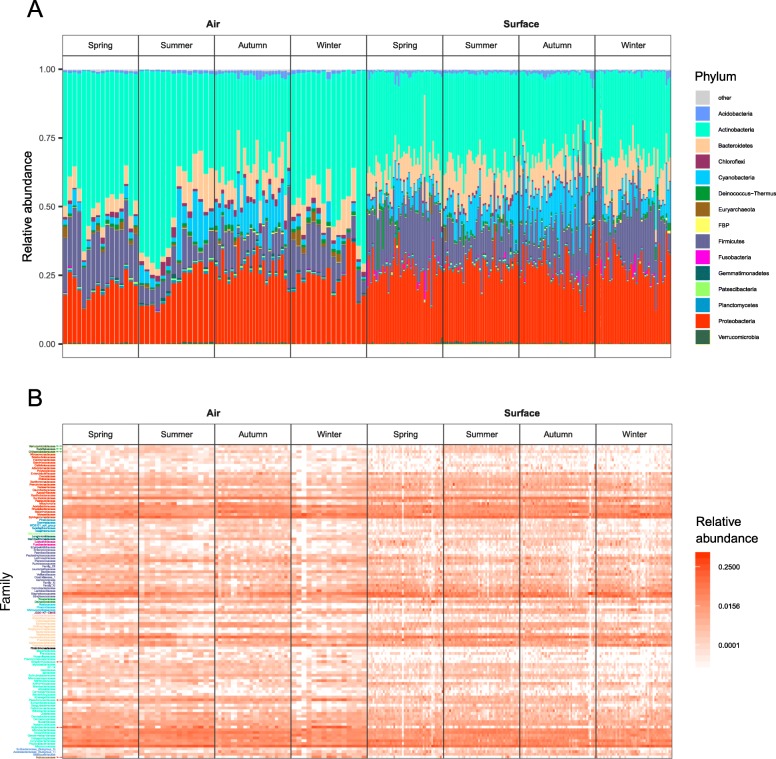
Taxonomic overview. **a** Relative abundances of the top 15 phyla. **b** Heatmap of most abundant families (relative abundance ≥ 0.01), color coded by phylum following the legend in panel **a**. Particularly differentiated features are highlighted with arrows, where green indicate seasonal variation and red variation between air and surface samples

In both air and surface samples, the phyla *Actinobacteria* and *Proteobacteria* dominated, with abundances of 42.9% and 23.9%, and 31.3% and 27.5% respectively (Table 1; Fig. 1a). The top 20 phyla were the same in both air and surface samples, with the top five also showing identical ordering by abundance. At the family level, *Micrococcaceae* was most abundant in both air and surface samples (10.5% and 7.2% respectively; Table 1). Notably, *Rubrobacteriaceae* and *Pseudonocardiaceae*, who were highly abundant in air samples (ranking as the 5th (3.5%) and 12th (2.1%) most abundant), were not found among the surface sample top 20 families, ranking as the 53rd (0.3%) and 48th (0.4%). The two unique families in the surface top 20 (*Lactobacillaceae*, *Deinococcaceae*) were both in the air top 25. At the genus level, similarities between the air and surface top 20 were still pronounced (Table 1). Most notably, *Staphylococcus* was the 2nd most abundant in air (3.8%) and most abundant in surface samples (4.7%). In line with the theme from the family level results, *Rubrobacter* (*Rubrobacteriaceae*), which ranked as the third most abundant in air (3.5%), was the 49th (0.3 %) most abundant in the surface samples. Of the other genera that appeared exclusively in the air top 20, only *Pseudonocardia* (1.1%) and *Nesterenkonia* (0.9%) had a substantially lower abundance ranking in surface (76th (0.2%) and 68th (0.2 %), respectively). Of the five genera that were exclusively in the surface top 20, only *Pseudomonas* ranked outside the air top 30 (37th).

**Table 1:** Top 20 phyla, families, and genera in air samples (*N* = 69) and surface samples (*N* = 177). Dots indicate that a group is also represented in the top 20 set from the other sample matrix. Prevalence is the sum of prevalence of all ASVs within a taxonomic group

Air samples				Surface samples			
Phylum		Prevalence	Abundance (%)	Phylum		Prevalence	Abundance (%)
*Actinobacteria*	•	32638	42.92	*Actinobacteria*	•	59693	31.32
*Proteobacteria*	•	27216	23.88	*Proteobacteria*	•	61648	27.47
*Firmicutes*	•	12494	11.97	*Firmicutes*	•	28790	16.65
*Bacteroidetes*	•	13390	8.10	*Bacteroidetes*	•	34972	10.99
*Cyanobacteria*	•	3988	6.29	*Cyanobacteria*	•	10775	7.88
*Chloroflexi*	•	2731	1.53	*Deinococcus-Thermus*	•	3184	1.37
*Acidobacteria*	•	2451	1.10	*Acidobacteria*	•	4904	1.05
*Deinococcus-Thermus*	•	1445	1.06	*Chloroflexi*	•	3591	0.67
*Planctomycetes*	•	1976	0.68	*Fusobacteria*	•	1557	0.59
*Euryarchaeota*	•	294	0.66	*Planctomycetes*	•	3321	0.51
*Gemmatimonadetes*	•	1335	0.53	*Verrucomicrobia*	•	2281	0.37
*Patescibacteria*	•	998	0.34	*Patescibacteria*	•	2086	0.33
*Verrucomicrobia*	•	910	0.33	*Gemmatimonadetes*	•	1667	0.26
*FBP*	•	410	0.17	*FBP*	•	958	0.18
*Armatimonadetes*	•	381	0.15	*Armatimonadetes*	•	773	0.12
*Fusobacteria*	•	250	0.10	*Euryarchaeota*	•	162	0.11
*Chlamydiae*	•	185	0.06	*Epsilonbacteraeota*	•	155	0.03
*Epsilonbacteraeota*	•	65	0.03	*Chlamydiae*	•	172	0.02
*Nitrospirae*	•	56	0.02	*Spirochaetes*	•	111	0.02
*Spirochaetes*	•	28	0.01	*Nitrospirae*	•	69	0.01
Family		Prevalence	Abundance (%)	Family		Prevalence	Abundance (%)
*Unassigned*	•	19657	15.01	*Unassigned*	•	42005	14.23
*Micrococcaceae*	•	3092	10.49	*Micrococcaceae*	•	5787	7.21
*Sphingomonadaceae*	•	3354	4.78	*Sphingomonadaceae*	•	8367	5.84
*Staphylococcaceae*	•	1965	4.26	*Staphylococcaceae*	•	4280	5.00
*Burkholderiaceae*	•	5034	4.06	*Streptococcaceae*	•	3810	4.53
*Rubrobacteriaceae*		1143	3.52	*Burkholderiaceae*	•	10578	4.42
*Hymenobacteraceae*	•	4187	3.35	*Hymebacteraceae*	•	9178	3.84
*Nocardioidaceae*	•	2899	3.24	*Moraxellaceae*	•	3982	3.35
*Moraxellaceae*	•	1689	2.96	*Corynebacteriaceae*	•	4365	3.34
*Acetobacteraceae*	•	2555	2.60	*Acetobacteraceae*	•	5957	2.64
*Corynebacteriaceae*	•	1578	2.49	*Cardioidaceae*	•	4933	2.29
*Intrasporangiaceae*	•	1498	2.30	*Propionibacteriaceae*	•	2947	2.20
*Pseudonocardiaceae*		1755	2.14	*Beijerinckiaceae*	•	3413	2.06
*Beijerinckiaceae*	•	1558	2.07	*Microbacteriaceae*	•	3654	1.93
*Geodermatophilaceae*	•	969	2.06	*Flavobacteriaceae*	•	4970	1.89
*Microbacteriaceae*	•	1604	1.80	*Intrasporangiaceae*	•	2498	1.52
*Propionibacteriaceae*	•	1224	1.71	*Geodermatophilaceae*	•	1747	1.46
*Streptococcaceae*	•	955	1.43	*Rhodobacteraceae*	•	3227	1.28
*Rhodobacteraceae*	•	1543	1.27	*Lactobacillaceae*		1474	1.16
*Flavobacteriaceae*	•	1476	1.13	*Deinococcaceae*		2604	1.15
Genus		Prevalence	Abundance (%)	Genus		Prevalence	Abundance (%)
*Unassigned*	•	38724	26.51	*Unassigned*	•	82052	24.72
*Micrococcus*	•	377	3.97	*Staphylococcus*	•	3208	4.57
*Staphylococcus*	•	1445	3.83	*Sphingomonas*	•	3184	4.23
*Rubrobacter*		1143	3.52	*Streptococcus*	•	2430	3.96
*Sphingomonas*	•	1260	3.37	*Hymenobacter*	•	8696	3.75
*Hymenobacter*	•	3850	3.19	*Corynebacterium*	•	3278	2.70
*Arthrobacter*	•	999	2.99	*Arthrobacter*	•	1904	2.07
*Corynebacterium*	•	1248	2.13	*Kocuria*	•	915	1.99
*Nocardioides*	•	1867	2.06	*Micrococcus*	•	594	1.82
*Psychrobacter*	•	453	1.49	*Psychrobacter*	•	1106	1.65
*Blastococcus*		494	1.38	*Flavobacterium*	•	3355	1.54
*Kocuria*	•	383	1.26	*Nocardioides*	•	2985	1.43
*Streptococcus*	•	658	1.22	*Cutibacterium*		523	1.23
*Pseudonocardia*		753	1.10	*Lactobacillus*	•	1459	1.16
*Nesterenkonia*		378	0.92	*Deinococcus*	•	2528	1.14
*Flavobacterium*	•	907	0.92	*Massilia*		1738	1.01
*Methylobacterium*		315	0.84	*Pseudomonas*		1545	0.92
*Deinococcus*	•	1025	0.80	*Acinetobacter*		1596	0.84
*Lactobacillus*	•	629	0.80	*1174-901-12*		1076	0.83
*Acidiphilium*	•	593	0.80	*Acidiphilium*	•	1432	0.78

**Fig. 2:**
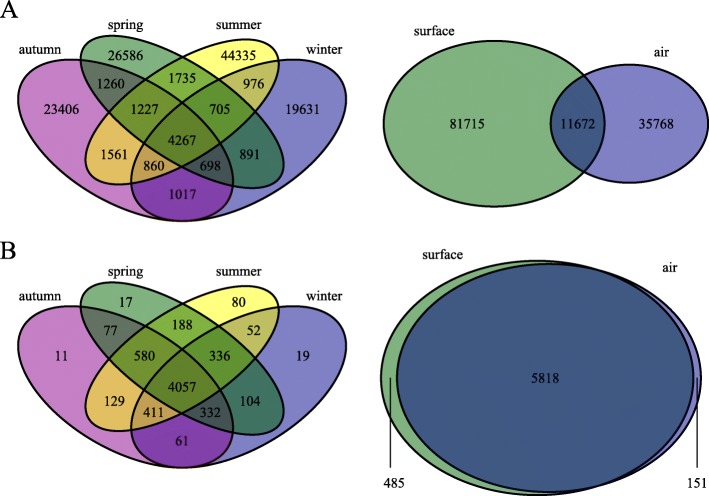
The distribution of amplicon sequence variants (ASVs) across seasons and sample matrices. Panel **a** includes all ASVs and panel **b** only ASVs with prevalence > 4 and abundance > 10

Abundance plots of phyla across seasons, and air and surface (Fig. 2a), showed a relatively stable distribution; however, *Firmicutes* exhibited higher relative abundance in winter and spring, while *Cyanobacteria* appeared to be more abundant during summer and autumn. As also seen in the top 20 abundance table (Table 1), *Actinobacteria* had a higher relative abundance in air samples, and *Proteobacteria* was more abundant in surface samples. We observed notable seasonal differentiation in three *Verrucomicrobia* families (*Verrucomicrobiaceae*, *Rubritaleaceae*, and *Chthoniobacteraceae*; Fig. 1b), who were all most abundant during summer, especially in surface samples. *Streptomycetaceae*, *Pseudonocardiaceae*, *Rubrobacteriaceae*, and *Halococcaceae* all showed higher relative abundance in air samples with no strong seasonal patterns (Fig. 1b).

The comparison of highly abundant taxa in surface samples taken from kiosks, benches, and railings revealed a high degree of similarity across surface types (Additional file 1: Table S5; Figure S5).

### Indicator genera: random forest classification

Random forest classification analyses, using genera as classification features, showed a high level of success in assigning samples to their correct bins (air or surface and correct season). The season classification had an out-of-bag error rate of 8.9%, with the highest class error found for summer samples, where nine samples were incorrectly classified as autumn samples (Table 2). *Psychrobacter* was the most important genus for correct season classification (MDA = 0.043, Table 2; Additional file 1: Figure S6).

**Table 2:** Random forest classification models of season

Season					
Out-of-bag estimate of error rate: 8.94%					
Confusion matrix	Autumn	Spring	Summer	Winter	Class error (%)
Autumn	53	1	5	1	11.7
Spring	0	54	1	1	3.6
Summer	9	0	63	0	12.5
Winter	2	2	0	54	6.9
Most important genera in sample classification					
Family:genera	Autumn	Spring	Summer	Winter	MDA
*Moraxellaceae*:*Psychrobacter*	0.026	0.039	0.040	0.069	0.043
*Microbacteriaceae*:*Cryobacterium*	0.022	0.057	0.011	0.004	0.022
*Flavobacteriaceae*:*Flavobacterium*	0.009	0.012	0.021	0.040	0.020
*Nocardioidaceae*:*Nocardioides*	0.020	0.024	0.011	0.013	0.016
*Flavobacteriaceae*:*Gillisia*	0.024	0.003	0.002	0.014	0.010
*Chitinophagaceae*:*Ferruginibacter*	0.009	0.012	0.007	0.009	0.009
*Gaiellaceae*:*Gaiella*	0.000	0.011	0.002	0.018	0.008
*Ilumatobacteraceae*:*CL500-29_marine_group*	0.001	0.015	0.003	0.012	0.007
*Burkholderiaceae*:*Polaromonas*	0.008	0.015	0.001	0.002	0.006
*Rubritaleaceae*:*Luteolibacter*	0.000	0.018	0.003	0.004	0.006
*Sphingomonadaceae*:*Qipengyuania*	0.001	0.001	0.009	0.010	0.005
*Clostridiaceae_1*:*Clostridium_sensu_stricto_13*	0.000	0.016	0.001	0.006	0.005
*Xanthomonadaceae*:*Thermomonas*	0.001	0.006	0.003	0.012	0.005
*Chthoniobacteraceae*:*Candidatus_Udaeobacter*	0.001	0.009	0.002	0.009	0.005
*Staphylococcaceae*:*Staphylococcus*	0.012	0.007	0.002	0.000	0.005
*Microbacteriaceae*:*Galbitalea*	0.003	0.017	0.000	0.001	0.005
*Pseudoalteromonadaceae*:*Pseudoalteromonas*	0.003	0.000	0.010	0.004	0.005
*Phormidiaceae*:*Tychonema_CCAP_1459:11B*	0.001	0.000	0.006	0.011	0.004
*Ilumatobacteraceae*:*Ilumatobacter*	0.001	0.006	0.002	0.009	0.004
*Demequinaceae*:*Demequina*	0.000	0.010	0.002	0.004	0.004

Confusion matrices show the classification of samples and the associated class error. The mean decrease in model accuracy (MDA; from removing the genus in question) and mean *Z*-scores are given for the 20 most important genera for classifying samples

The classification of samples as either air or surface had an out-of-bag error rate of 6.1%. The model was highly successful in correctly classifying surface samples, with only two samples being wrongly assigned as air samples (class error = 1.1%). Air samples, on the other hand, had a substantial class error (18.8%), with 13 of 69 air samples being classified as surface samples (Table 3). *Ralstonia* was the most important genus for correct classification (MDA = 0.015; Table 3; Additional file 1: Figure S7).

**Table 3:** Random forest classification models of air/surface

Air/surface			
Out-of-bag estimate of error rate: 6.1%			
Confusion matrix	Air	Surface	Class error (%)
Air	56	13	18.8
Surface	2	175	1.1
Most important genera in sample classification			
Family:Genera	Air	Surface	MDA
*Burkholderiaceae:Ralstonia*	0.027	0.010	0.015
*Streptomycetaceae:Streptomyces*	0.020	0.006	0.010
*Pseudonocardiaceae:Pseudonocardia*	0.018	0.006	0.009
*Streptococcaceae:Streptococcus*	0.015	0.004	0.007
*Pseudonocardiaceae:Saccharopolyspora*	0.012	0.004	0.006
*Neisseriaceae:Neisseria*	0.013	0.003	0.006
*Nocardiopsaceae:Nocardiopsis*	0.011	0.004	0.006
*Rubrobacteriaceae:Rubrobacter*	0.011	0.004	0.006
*Micrococcaceae:Micrococcus*	0.008	0.004	0.005
*Carnobacteriaceae:Granulicatella*	0.012	0.003	0.005
*Pasteurellaceae:Haemophilus*	0.012	0.002	0.005
*Peptostreptococcaceae:Terrisporobacter*	0.008	0.004	0.005
*Micrococcaceae:Pseudarthrobacter*	0.009	0.003	0.005
*Planococcaceae:Planomicrobium*	0.009	0.003	0.005
*Halococcaceae:Halococcus*	0.007	0.003	0.004
*Micrococcaceae:Rothia*	0.008	0.002	0.004
*Halococcaceae:Halalkalicoccus*	0.007	0.002	0.003
*Porphyromonadaceae:Porphyromonas*	0.007	0.002	0.003
*Planococcaceae:Planococcus*	0.007	0.002	0.003
*Pseudonocardiaceae:Actinomycetospora*	0.006	0.002	0.003

Confusion matrices show the classification of samples and the associated class error. The mean decrease in model accuracy (MDA; from removing the genus in question) and mean *Z*-scores are given for the 20 most important genera for classifying samples

The classification analysis of samples by surface type had a substantially higher out-of-bag error rate (42.37%; Additional file 1: Table S6), with a large number of samples being misclassified for all surface types. The error was particularly pronounced for railing samples, where 41 out of 56 samples were not binned correctly (class error of 73.2%).

### Diversity

When assessing all ASVs without prevalence or abundance filtering, we found the majority to be exclusive to one season, and either surface or air (Fig. 2a). On the other hand, ASVs that had prevalence > 4 and abundance > 10 were largely present across all seasons and in both surface and air samples (Fig. 2b).

**Fig. 3:**
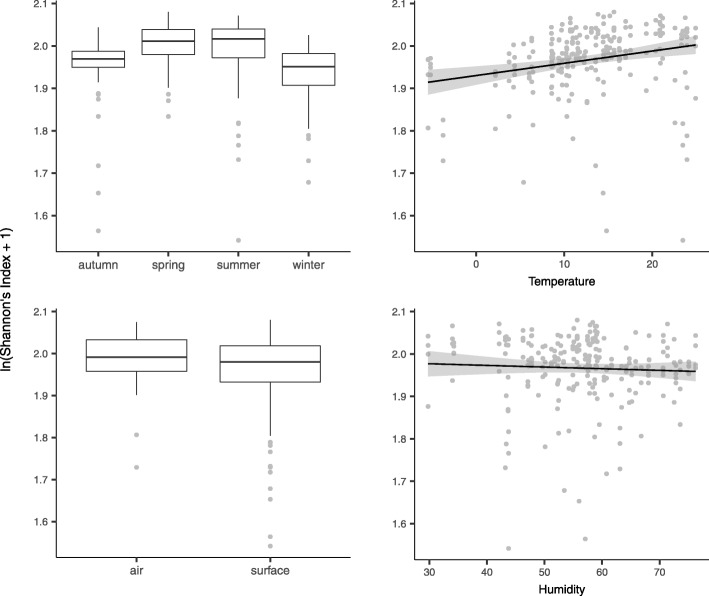
Analysis of Shannon’s diversity index. The four significant predictors of within-sample diversity (see Table 4)

For Shannon’s diversity index scores, the models that assessed variables specific to surface (surface type, material, and treatment) were all non-significant (all *p* > 0.16). The step-wise AIC model selection scheme on a model with the remaining predictors—air/surface, season, indoor/outdoor station, time of day, temperature, humidity, sequence run, and all possible two-way interactions—returned a model which contained four significant predictors (temperature, *p* < 0.001; air/surface, *p* = 0.005; season, *p* < 0.001; and humidity, *p* = 0.017; Fig. 3) and four significant interactions, which together explained 27% of the variance in Shannon’s diversity index scores and had an overall *p* value of 1.04 × 10^−09^ (Table 4). Diversity was higher during spring and summer, in air samples, and at higher temperatures and lower levels of humidity (Fig. 3). Of the significant interaction effects, temperature: air/surface (*p* = 0.002) was most notable; closer inspection indicated that surface samples had higher diversity than air samples at low temperatures, and lower diversity at higher temperatures (Additional file 1: Figure S8).

**Table 4:** The best-fit model for Shannon’s diversity index score, which explained 27% of within-sample diversity variance and had a *p* value of 1.04 × 10^−09^. Slopes are given for continuous predictor variables and interactions between continuous and categorical predictors with two levels. Observed trends, from low to high average Shannon’s diversity scores, are given for the categorical predictors

Predictor	DF	Sum Sq.	Mean Sq.	*F*	*p*	Slope/trend
Temperature	1	0.089	0.089	17.62	< 0.001	0.0006
Air/surface	1	0.041	0.041	8.20	0.005	Surface > air
Season	3	0.102	0.034	6.75	< 0.001	Winter > autumn > summer > spring
Humidity	1	0.029	0.029	5.74	0.017	− 0.0017
Humidity SD	1	0.005	0.005	1.04	0.309	− 0.1415
Temperature SD	1	0.002	0.002	0.47	0.493	− 0.1018
Time of day	1	0.002	0.002	0.43	0.514	− 0.0001
Indoor/Outdoor	1	0.000	0.000	0.000	1.000	Outdoor > indoor
Temperature SD: temperature	1	0.097	0.097	19.11	< 0.001	− 0.0130
Temperature: air/surface	1	0.051	0.051	10.02	0.002	− 0.0047
Time: indoor/outdoor	1	0.029	0.029	5.75	0.017	0.0005
Humidity SD: season	3	0.061	0.020	4.04	0.008	
Time: season	3	0.034	0.011	2.24	0.085	
Season: indoor/outdoor	3	0.029	0.010	1.91	0.130	
Humidity SD: humidity	1	0.005	0.005	1.01	0.316	0.0011
Temperature SD: season	3	0.006	0.002	0.37	0.773	
Humidity SD: temperature	1	0.000	0.000	0.08	0.772	0.0060
Temperature SD: time of day	1	0.000	0.000	0.00	0.990	0.0004
Residuals	209	1.056	0.005			

**Fig. 4:**
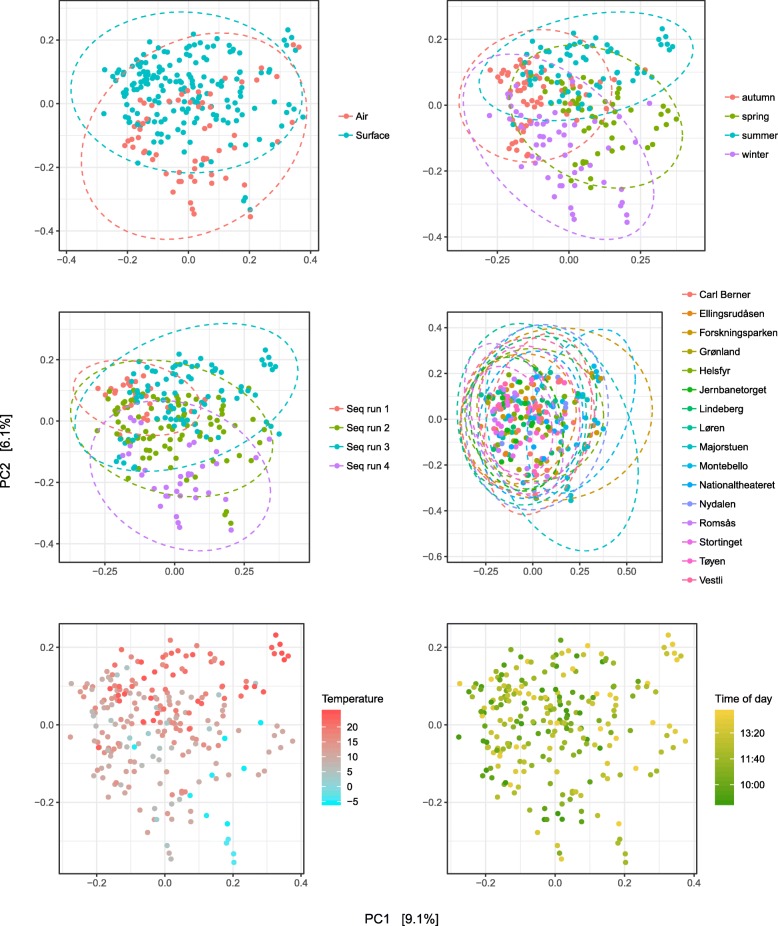
Analysis of Bray–Curtis dissimilarity distances. PCoA plots of among-sample diversity with significant predictors from the PERMANOVA model (see Table 5). Dashed circles represent 95% CI for each cluster

In a multivariate PERMANOVA model of among-sample diversity (ordinated Bray–Curtis dissimilarity) with predictors specific to surface, we found only surface type to be significant (*F* = 2.03, *R*^2^ = 0.02, *p* = 0.001; Additional file 1: Figure S9). A PERMANOVA model with the remaining predictors (air/surface, season, indoor/outdoor station, time of day, temperature, humidity, sequence run, and all possible two-way interactions) was subjected to a step-wise AIC model selection scheme, which produced a model that explained 56% of among-sample diversity. This model included six predictors, and three two-way interactions (Table 5). Whether samples were taken from air or surface was a highly significant predictor (*p* = 0.001), explaining 4% of the total variance. Season (*p* = 0.001) and subway station (*p* = 0.001) explained 11%, and 15% of the variance respectively. Sequence run was also a significant predictor of among-sample diversity (*p* = 0.001) and explained 2% of the variance. Ordination plots revealed clear differentiation for air/surface, season, and sequence run (Fig. 4); however, subway stations, which explained the largest amount of variance, showed no discernable clustering.

**Table 5:** The best-fit PERMANOVA model, which explained 56% of among-sample diversity (Bray–Curtis dissimilarity)

Predictor	DF	Sum Sq.	Mean Sq.	*F*	*R*^2^	*p*
Air/surface	1	2.484	2.484	14.16	0.04	0.001
Season	3	6.593	2.198	12.52	0.11	0.001
Subway station	15	9.134	0.609	3.47	0.15	0.001
Temperature	1	0.475	0.475	2.71	0.01	0.001
Sequence run	3	1.063	0.354	2.02	0.02	0.001
Time of day	1	0.239	0.239	1.36	< 0.01	0.050
Season: air/surface	3	0.853	0.284	1.620	0.01	0.001
Subway station: air/surface	15	3.382	0.225	1.28	0.06	0.001
Season: subway station	44	9.792	0.223	1.27	0.16	0.001
Residuals	151	26.500	0.175		0.44

## Discussion

Mass transit systems are BEs critical to the everyday lives of a vast number of people, and its potential role in the transmission of infectious diseases as well as bioterrorism risk cannot be understated [6, 12, 13]. With the advent of molecular assay techniques—more recently high-throughput sequencing—we are no longer restricted to culture-based techniques and can better unravel microbial diversity in mass transit systems. Characterization of microbial diversity in such environments is vital to understand the dynamics of antimicrobial resistance and enables the detection and monitoring of potential pathogens and bioterrorism threat agents. Furthermore, it is essential for the understanding of how our own microbiomes interact with the microbiomes that surround us, and how this ultimately may affect our health and wellbeing [3, 9]. A vital step in this effort is to explore the variability of mass transit microbiomes across sample matrices and temporal scales, and identify important drivers of such variation. In this study, we described the biological background in both air and surfaces from 16 subway stations in Oslo, Norway—a smaller and more northerly city compared with other cities where subway microbiomes have previously been mapped. We provide a direct comparison of surface and air communities, and an assessment of seasonal variation in subway microbiomes.

### Taxonomy, relative abundances, and ecology

In the entire dataset, over 300 K unique ASVs were identified. This is substantially higher than comparable studies [5, 7, 20, 21]; however, direct comparisons of studies are not feasible since differences in sampling and wet lab protocols, and sequencing depths may strongly influence results. Further, the use of different taxonomic classifiers with different sensitivities will have substantial effects on the number of OTUs/ASVs reported [39].

The top five most abundant phyla in both surface and air samples (Table 1; Fig. 1a) matched the top five phyla in the Mexico City subway (station and train surfaces) [7] perfectly, the only difference being their ordering by relative abundance. Further, three genera in the top five overlapped between our surface samples and the Mexico City study: *Staphylococcus*, *Streptococcus*, *Corynebacterium*. Major phyla identified in the subway studies from Hong Kong subway [20] and New York [21] were also the same as those identified in the present study.

We found that many less abundant ASVs were unique to specific seasons or sample matrices, while abundant groups were, for the most part, ubiquitous across seasons, and surface and air samples; importantly, a very low filtering cutoff was sufficient to remove almost all taxa only present in single seasons or a single sample matrix (Fig. 2). In surface and air samples, the top 20 most abundant phyla were the same and ordered identically. Two families were highly abundant in air but not in surface samples: *Rubrobacteriaceae* and *Pseudonocardiaceae* with relative abundances of 3.52% and 2.14% in air samples. Our findings were similar at the genus level: the radiotolerant [40] *Rubrobacter* (which constituted the *Rubrobacteriaceae* contribution in its entirety; 3.52%) had a uniquely high abundance in air, along with *Pseudonocardia* (most notably producers of antibiotics for use in pest control by fungus farming ants [41]; 1.10%), and *Nesterenkonia* (ubiquitous, and in extreme environments, opportunistically pathogenic [42] 0.92%). We have no explanation for why these particular bacteria were so abundant in air, but not on surfaces. One might expect that all bacteria in air eventually settle on surfaces; however, the chemical and biological properties, and size of bacteria [43], along with environmental variables in air, can affect both deposition and resuspension rates, which introduces a high level of complexity in the relationship between air and surface microbiomes.

We observed that three *Verrucomicrobia* families (*Verrucomicrobiaceae*, *Rubritaleaceae*, and *Chthoniobacteraceae*) varied in abundance across seasons, showing the highest abundance during summer (Fig. 1b). *Verrucomicrobia*, which is part of the PVC superphylum, is ecologically diverse, often highly abundant and present in a range of different environments [44].

Among the three investigated surface types—kiosks, benches, and railings—we found more congruency among the highly abundant taxa (Additional file 1: Table S5), as compared with the level of differentiation observed between air and surface (Table 1).

To identify genera that were highly divergent among seasons, surface and air, and surface types, we performed random forest classification analyses, where genera were scored by their ability to bin samples in their correct category (season/sample matrix/surface type). The two genera with the highest importance for classifying samples by season, namely *Psychrobacter*, and *Cryobacterium* (Table 2) are both psychrophilic (cold tolerance or preference towards colder temperatures) [45, 46]. *Psychrobacter* was most abundant during winter and *Cryobacterium* during spring (Table 2; Additional file 1: Figure S6). For correctly binning surface and air samples, *Ralstonia* and *Streptomyces* were the most important genera, both being more abundant in air samples (Table 3; Additional file 1: Figure S7). *Ralstonia* are environmental opportunistically pathogenic bacilli [47], while *Streptomyces* is a species-rich genus, highly abundant in soil where they play an important role in carbon cycling [48]. We note that *Ralstonia* has been identified as a common contaminant in sequence library preparation steps [49] and that such contaminants may introduce stronger bias in sequence data from low-biomass samples, such as air [50]. The random forest classification of samples by surface type performed very poorly (Additional file 1: Table S6), which indicated that genus level taxonomic composition is not strongly diverged among surface types. Thus, we conclude that taxonomic representation is much more similar across surface types, than across air/surface or different seasons.

### Diversity

Analyses of within-sample diversity (Shannon’s diversity index) and among-sample diversity (ordinated Bray–Curtis dissimilarity distances) revealed several interesting patterns. We analyzed diversity with some hitherto untried predictors (season, surface/air, indoor/outdoor stations), and some that have been included in previous subway studies (temperature, humidity, time of day, surface types).

We found no evidence for within-sample diversity differing among surface types (kiosks, railings, and benches). Analysis of among-sample diversity *did* reveal a significant association (Additional file 1: Figure S9), although only a relatively small proportion of the variance in among-sample diversity was explained by surface type (~ 2%). This latter finding is congruent with that of Hsu et al. [5], who found that microbial community structure varied significantly across surface types in the Boston metropolitan transit system.

Previous studies have found time of day to be a highly important variable for understanding subway microbiome fluctuations, with peak and non-peak commuting hours showing marked differences [15, 17]. We found time of day to be a significant predictor of among-sample diversity (Table 5; Fig. 4), but that it explained a relatively small proportion of the total variance in diversity, as compared with the other predictors. This may partly be due to the huge difference in number of commuters between Oslo, and Hong Kong and Beijing, and that the present study sampled outside peak commuting hours. Furthermore, the study design used here is not suited to properly gauge the importance of time of day—since this would require within-day repeated sampling for single locations.

Temperature was a highly significant predictor of both within-sample (Table 4; Fig. 3) and among-sample diversity (Table 5; Fig. 4), whereas humidity was only significant for within-sample diversity (Table 4; Fig. 3). Note that a very small proportion of the among-sample diversity total variance was explained by temperature (Table 5), while the effect size on within-sample diversity was pronounced (Fig. 3). Leung et al. [20] also found temperature and humidity to influence microbial diversity in the Hong Kong subway; note however, these associations were only significant when including outdoor stations. Our results show that humidity had a weak negative impact on diversity (Fig. 3), which is not congruent with Leung et al. who found a positive association. This incongruity may be explained by the variability and non-linear nature of the association between humidity and bacterial survival rates [51], which may give rise to different results across geographical areas and temporal scales. Humidity ranged from approximately 50 to 80% in the Leung et al. study, while our data ranged from 29.8 to 76.3%. Leung et al. found a negative association between temperature and diversity, the opposite of what we observe. Again, this is perhaps explained by the lack of overlap in the temperature range in the two studies (Leung et al.: approximately 24–30 °C; our study: − 5.45–24.91 °C).

Three of the 16 stations included in this study were outdoor subway stations. Indoor/outdoor was borderline significant in a univariate test (*p* = 0.08) of within-sample diversity; however, there was no significant association in the final multivariate model. The temperatures at outdoor stations will vary significantly throughout the seasons and even throughout the day, which may drive the (nearly significant) association between indoor/outdoor and within-sample diversity. When removing temperature from the final model of within-sample diversity, indoor/outdoor was again borderline significant (*p* = 0.07), which leads us to conclude that temperature outcompetes indoor/outdoor in our model (Table 4). Much like for temperature, we found indoor/outdoor to be a significant predictor of diversity in air samples (univariate; *p* = 0.04), but not in surface samples (univariate; *p* = 0.29). Reiterating the observed dynamic between indoor/outdoor and temperature mentioned above, a model with indoor/outdoor and temperature as predictors of air sample diversity only supported temperature (*p =* 0.23, *p =* 5 × 10^−10^, respectively). Although outdoor air is known to be a major source for indoor microbiomes [25], one would expect commuters, another important source [20], to be a more significant contributor in indoor environments. Hence, the lack of significance in univariate tests of indoor/outdoor as a predictor of diversity is an unexpected finding. One possible explanation is that there are relatively few commuters in Oslo, making human sources less dominant, or that effective air exchange reduces the differences between indoor and outdoor air.

A major aim of this study was to compare subway air and surface microbiomes, and we found air/surface to be a highly significant predictor of both within-sample and among-sample diversity (Tables 4 and 5; Figs. 3 and 4). Importantly, the effect of this association was dependent on temperature; we found air to have lower within-sample diversity at low temperatures, and higher diversity at high temperatures (Additional file 1: Figure S8). This can be explained by microbial diversity in air being more sensitive to temperature, as compared with surface. To evaluate this hypothesis, we ran post hoc univariate analyses of Shannon’s diversity index scores and temperature on air and surface samples separately, which found temperature to be a non-significant predictor for surface samples, (*R*^2^ = 0.01; *p* = 0.08), but highly significant for air samples (*R*^2^ = 0.52; *p* = 4.05 × 10^−11^). It appears that the diversity differences in air and surface microbiomes to a large extent are driven by differential effects of temperature. One explanation for this observation is the association between temperature and air circulation regimes, which can strongly influence air microbiome composition [52].

We found significant differences in within-sample and among-sample diversity across seasons (Tables 4 and 5; Figs. 3 and 4). Within-sample diversity was highest during spring and summer (Fig. 3). Apart from subway station, seasons explained the largest amount of among-sample diversity of all included predictors (*R*^2^ = 0.11; Table 5). Seasonal variation has not previously been evaluated in subways using culture-independent methods; however, Patel et al. [28] cultured bacteria and fungi from dust collected at railway stations in England and Scotland, and Heo et al. [14] measured concentrations of culturable bacteria in underground subway stations through spring and autumn. Both studies are congruent with the results presented here; bacterial numbers increased from spring through summer and decrease towards winter. Several studies have observed seasonality in atmospheric microbiome composition [22–24]. With the outdoors being an important source for BE microbiomes [25], this suggests that seasonal variations in subway microbiomes may be influenced, at least partly, by seasonal changes in atmospheric microbial communities.

Subway station was a highly significant predictor of among-sample diversity, explaining 15% of the total variance (Table 5). However, when inspecting the clustering of PCoA ordinated values in Fig. 4, there are no clear patterns. We suspect that this result is mainly a consequence of including a categorical predictor with too many levels. Hence, we must refrain from concluding on the importance of subway station as a predictor of microbiome composition in our study. Sequence run was also a significant predictor of among-sample diversity and explained 2% of the total variance. We propose that this stems from an unbalanced partitioning of samples from different seasons, sample matrices, or other variables into the four sequence runs. Alternatively, the association with sequence run may be explained by predictors not included. Both these explanations are congruent with the qPCR results, which show higher bacterial load in the samples from sequence run 3 (Additional file 1: Table S4 and Figure S2).

### Caveats

In our study, seasonality was assessed by sampling on single days within seasons without accounting for the variation in shorter time periods (e.g., weekly variation) or repeatability across years. While patterns such as differential abundance of certain taxa in spring and summer, compared with autumn and winter are convincing, a higher resolution sampling scheme should be implemented in the future to distinguish between variations that occur on different timescales. Although we provide a relatively high level of geographical resolution in the present study, we recommend that future studies address seasonal and air/surface variability across cities, countries, and continents using standardized methods.

## Conclusions

Understanding the composition and dynamics of air and surface microbiomes in mass transit environments—given their role in facilitating interactions among human and other BE microbiomes as well as infectious disease transmission and bioterrorism risk—is important for future developments in human health and security. Here we provide increased resolution to the state-of-knowledge regarding subway microbiomes by showing that there are significant differences between air and surface microbiomes, identifying seasonality as a central driver of subway microbiome variability, and confirming patterns previously observed in different geographical and demographical contexts. These results imply that biological detection, identification, and monitoring efforts in BEs should take into consideration the different characteristics/properties of air and surfaces, and that studies of microbial community composition should include seasonal sampling.

## Supplementary information

**Additional file 1: Table S1.** Type of environment, latitude and longitude for all sampled stations. **Table S2.** Overview of all samples included in the analyses. **Table S3.** PCR program for 16S rRNA gene amplicon sequencing. **Table S4.** The best-fit models of qPCR 16S rRNA gene copies for air samples and surface samples. **Table S5.** Top 20 phyla, families, and genera and species in surface samples collected on kiosks, benches, and railings. **Table S6.** Random forest classification models of samples collected from different surface types. **Figure S1.** The significant predictors of qPCR 16S rRNA gene copy yields in air samples. **Figure S2.** The significant predictors of qPCR 16S rRNA gene copy yields in surface samples. **Figure S3.** Quality profile of filtered reads. **Figure S4.** Rarefaction curves with observed diversity and Shannon’s Diversity Index. **Figure S5.** **A)** Relative abundances of the top 15 phyla across the three surface types and seasons. **B**) Heatmap of most abundant families. **Figure S6.** Top 20 most important genera in random forest classification analysis of samples collected in different seasons. **Figure S7.** Top 20 most important genera in random forest classification analysis of air and surface samples. **Figure S8.** Interaction effect between temperature (°C) and air/surface in the linear model of Shannon’s diversity index. **Figure S9.** PCoA plot of Bray Curtis dissimilarity distances with the only significant predictor (surface type) from the PERMANOVA model that included only surface-specific predictors.

## Data Availability

The sequence data have been deposited in the NCBI Sequence Read Archive under accession PRJNA566330. (https://www.ncbi.nlm.nih.gov/sra/PRJNA566330).
