# Supplementary material for: The subway microbiome: seasonal dynamics and direct comparison of air and surface bacterial communities
Source: Microbiome. 2019 Dec 19;7:160. doi: 10.1186/s40168-019-0772-9 (PMC6924074; doi:10.1186/s40168-019-0772-9)

Supplementary information for:

The subway microbiome: Seasonal dynamics and direct comparison of air and surface bacterial communities.

Authors:

Jostein Gohli[1*], Kari Oline Bøifot[1,2], Line Victoria Moen[1], Paulina Pastuszek[3], Gunnar Skogan[1], Klas I. Udekwu[4], and Marius Dybwad[1,2]

1) Norwegian Defence Research Establishment, P O Box 25, NO-2027 Kjeller, Norway

2) Department of Analytics, Environmental & Forensic Sciences, King's College London, 150 Stamford Street, London SE1 9NH, UK

3) Department Of Molecular Biosciences, Stockholm University, TWGI Stockholm, SE 10691

4) SoS BIOs Sweden, Tiundagatan 41, Uppsala SE 752 30

Table S1 – Type of environment, latitude and longitude for all sampled stations.

Station	Environment	Latitude	Longitude
Carl Berners plass	Indoor	59.92566	10.77959
Ellingsrudåsen	Indoor	59.93651	10.91664
Grønland	Indoor	59.91278	10.76009
Helsfyr	Indoor	59.91259	10.80139
Jernbanetorget	Indoor	59.91226	10.75027
Lindeberg	Indoor	59.93276	10.88167
Løren	Indoor	59.93010	10.78959
Nationaltheateret	Indoor	59.91546	10.73209
Nydalen	Indoor	59.94940	10.76592
Romsås	Indoor	59.96260	10.89248
Stortinget	Indoor	59.91343	10.74329
Tøyen	Indoor	59.91558	10.77580
Vestli	Indoor	59.97204	10.92944
Forskningsparken	Outdoor	59.94370	10.72088
Majorstuen	Outdoor	59.93016	10.71473
Montebello	Outdoor	59.93482	10.66640

Table S2 - Overview of all samples included in the analyses.

Season	Air samples (n)	Surface samples (n)	Collection date
Autumn	16	44	17.nov.16
Winter	15	43	07.feb.17
Spring	14	42	20.apr.17
Summer	24	48	28.jun.17

Table S3 - PCR program for 16S rRNA gene amplicon sequencing.

	Temperature (°C)	Time	Cycles
Denaturation	98	30 sec	1
Denaturation	98	10 sec	30
Annealing	55	30 sec	
Extension	72	30 sec	
Final extension	72	4 min	1
Hold	4		

Table S4 – The best-fit models of qPCR 16S rRNA gene copies for A) air samples and B) surface samples.

**A**						
Predictor	*Df*	*Sum Sq*	*Mean Sq*	*F*	*P*	
Humidity SD	1	0.46	0.46	0.55	0.461	
Humidity	1	7.02	7.02	8.52	**0.005**	**
Season	3	14.34	4.78	5.80	**0.002**	**
Residuals	55	45.33	0.82			
**B**						
Predictor	*Df*	*Sum Sq*	*Mean Sq*	*F*	*P*	
Sequence run	2	49.51	24.75	27.25	**<0.001**	***
Humidity SD	1	1.44	1.44	1.59	0.209	
Temperature	1	0.78	0.78	0.86	0.355	
Humidity	1	2.21	2.21	2.43	0.121	
Season	3	15.18	5.06	5.57	**0.001**	**
Indoor/Outdoor	1	17.34	17.34	19.09	**<0.001**	***
Surface type	2	48.91	24.46	26.92	**<0.001**	***
Residuals	164	148.96	0.91			

Table S5 - Top 20 phyla, families, and genera and species in surface samples collected on kiosks (N=62), benches (N=59), and railings (N=56). Dots indicate that a group is also represented in the top 20 set from the other two surface types.

Kiosk samples			*Bench samples*			*Railing samples*		
**Phylum**		**abundance**	**Phylum**		**abundance**	**Phylum**		**abundance**
Actinobacteria	•	32.40 %	*Actinobacteria*	•	31.94 %	*Actinobacteria*	•	29.51 %
Proteobacteria	•	26.73 %	*Proteobacteria*	•	28.28 %	*Proteobacteria*	•	27.35 %
Firmicutes	•	18.26 %	*Firmicutes*	•	13.98 %	*Firmicutes*	•	17.91 %
Bacteroidetes	•	9.96 %	*Bacteroidetes*	•	11.04 %	*Bacteroidetes*	•	12.02 %
Cyanobacteria	•	6.74 %	*Cyanobacteria*	•	9.60 %	*Cyanobacteria*	•	7.19 %
Deinococcus-Thermus	•	1.60 %	*Deinococcus-Thermus*	•	1.31 %	*Deinococcus-Thermus*	•	1.19 %
Acidobacteria	•	1.00 %	*Acidobacteria*	•	1.05 %	*Acidobacteria*	•	1.11 %
Chloroflexi	•	0.66 %	*Chloroflexi*	•	0.67 %	*Fusobacteria*	•	0.80 %
Fusobacteria	•	0.55 %	*Planctomycetes*	•	0.46 %	*Chloroflexi*	•	0.68 %
Planctomycetes	•	0.50 %	*Fusobacteria*	•	0.44 %	*Planctomycetes*	•	0.56 %
Verrucomicrobia	•	0.40 %	*Verrucomicrobia*	•	0.32 %	*Patescibacteria*	•	0.39 %
Patescibacteria	•	0.32 %	*Patescibacteria*	•	0.29 %	*Verrucomicrobia*	•	0.39 %
Gemmatimonadetes	•	0.29 %	*Gemmatimonadetes*	•	0.22 %	*Gemmatimonadetes*	•	0.27 %
Euryarchaeota	•	0.19 %	*FBP*	•	0.17 %	*FBP*	•	0.20 %
FBP	•	0.18 %	*Armatimonadetes*	•	0.11 %	*Armatimonadetes*	•	0.14 %
Armatimonadetes	•	0.13 %	*Epsilonbacteraeota*	•	0.03 %	*Euryarchaeota*	•	0.13 %
Epsilonbacteraeota	•	0.02 %	*Euryarchaeota*	•	0.02 %	*Epsilonbacteraeota*	•	0.04 %
Chlamydiae	•	0.02 %	*Chlamydiae*	•	0.02 %	*Chlamydiae*	•	0.03 %
Spirochaetes	•	0.01 %	*Spirochaetes*	•	0.01 %	*Spirochaetes*	•	0.02 %
Fibrobacteres		0.01 %	*WPS-2*		0.01 %	*Nitrospirae*		0.01 %
**Family**		**abundance**	**Family**		**abundance**	**Family**		**abundance**
Unassigned	•	12.72 %	*Unassigned*	•	16.64 %	*Unassigned*	•	13.16 %
Micrococcaceae	•	7.75 %	*Micrococcaceae*	•	7.33 %	*Micrococcaceae*	•	6.51 %
Staphylococcaceae	•	5.88 %	*Sphingomonadaceae*	•	6.32 %	*Staphylococcaceae*	•	5.47 %
Sphingomonadaceae	•	5.80 %	*Burkholderiaceae*	•	4.95 %	*Streptococcaceae*	•	5.37 %
Corynebacteriaceae	•	4.17 %	*Streptococcaceae*	•	4.49 %	*Sphingomonadaceae*	•	5.34 %
Burkholderiaceae	•	4.05 %	*Hymenobacteraceae*	•	4.33 %	*Burkholderiaceae*	•	4.23 %
Streptococcaceae	•	3.77 %	*Staphylococcaceae*	•	3.75 %	*Moraxellaceae*	•	4.06 %
Hymenobacteraceae	•	3.39 %	*Corynebacteriaceae*	•	2.76 %	*Hymenobacteraceae*	•	3.78 %
Moraxellaceae	•	3.32 %	*Moraxellaceae*	•	2.75 %	*Corynebacteriaceae*	•	3.12 %
Acetobacteraceae	•	2.71 %	*Acetobacteraceae*	•	2.73 %	*Acetobacteraceae*	•	2.46 %
Propionibacteriaceae	•	2.37 %	*Nocardioidaceae*	•	2.38 %	*Flavobacteriaceae*	•	2.37 %
Nocardioidaceae	•	2.14 %	*Beijerinckiaceae*	•	2.29 %	*Nocardioidaceae*	•	2.37 %
Beijerinckiaceae	•	2.02 %	*Propionibacteriaceae*	•	2.24 %	*Propionibacteriaceae*	•	1.98 %
Microbacteriaceae	•	1.69 %	*Microbacteriaceae*	•	2.21 %	*Microbacteriaceae*	•	1.89 %
Lactobacillaceae		1.58 %	*Flavobacteriaceae*	•	1.92 %	*Beijerinckiaceae*	•	1.84 %
Deinococcaceae		1.42 %	*Intrasporangiaceae*	•	1.73 %	*Prevotellaceae*		1.52 %
Flavobacteriaceae	•	1.39 %	*Geodermatophilaceae*		1.72 %	*Intrasporangiaceae*	•	1.48 %
Family_XI		1.37 %	*Rhodobacteraceae*		1.46 %	*Geodermatophilaceae*		1.34 %
Intrasporangiaceae	•	1.35 %	*Deinococcaceae*		1.14 %	*Rhodobacteraceae*		1.21 %
Pseudomonadaceae		1.34 %	*Pseudomonadaceae*		1.03 %	*Lactobacillaceae*		1.19 %
**Genus**		**abundance**	**Genus**		**abundance**	**Genus**		**abundance**
Unassigned	•	22.58 %	*Unassigned*	•	28.00 %	*Unassigned*	•	23.36 %
Staphylococcus	•	5.56 %	*Sphingomonas*	•	4.58 %	*Streptococcus*	•	4.90 %
Sphingomonas	•	4.31 %	*Hymenobacter*	•	4.21 %	*Staphylococcus*	•	4.80 %
Corynebacterium	•	3.43 %	*Streptococcus*	•	3.87 %	*Sphingomonas*	•	3.76 %
Hymenobacter	•	3.32 %	*Staphylococcus*	•	3.43 %	*Hymenobacter*	•	3.68 %
Streptococcus	•	3.16 %	*Arthrobacter*	•	2.54 %	*Corynebacterium*	•	2.56 %
Kocuria	•	2.65 %	*Corynebacterium*	•	2.13 %	*Flavobacterium*	•	2.00 %
Micrococcus	•	2.22 %	*Kocuria*	•	1.85 %	*Psychrobacter*	•	1.83 %
Arthrobacter	•	1.81 %	*Psychrobacter*	•	1.52 %	*Micrococcus*	•	1.82 %
Psychrobacter	•	1.60 %	*Flavobacterium*	•	1.48 %	*Arthrobacter*	•	1.81 %
Lactobacillus		1.57 %	*Nocardioides*	•	1.47 %	*Nocardioides*	•	1.49 %
Cutibacterium	•	1.43 %	*Micrococcus*	•	1.43 %	*Kocuria*	•	1.46 %
Deinococcus	•	1.41 %	*Massilia*	•	1.22 %	*Lactobacillus*		1.19 %
Nocardioides	•	1.33 %	*Deinococcus*	•	1.13 %	*Cutibacterium*	•	1.15 %
Pseudomonas		1.20 %	*Cutibacterium*	•	1.12 %	*Acinetobacter*		1.14 %
Flavobacterium	•	1.17 %	*1174-901-12*		1.03 %	*Massilia*	•	0.91 %
Massilia	•	0.89 %	*Pseudomonas*		0.91 %	*Haemophilus*		0.91 %
1174-901-12		0.79 %	*Roseomonas*		0.86 %	*Deinococcus*	•	0.88 %
Methylobacterium		0.79 %	*Blastococcus*		0.83 %	*Acidiphilium*		0.81 %
Pedobacter		0.76 %	*Nakamurella*		0.82 %	*Veillonella*		0.75 %

Table S6 - Random forest classification models of samples collected from different surface types. Confusion matrices show the classification of samples and the associated class error. The mean decrease in model accuracy (MDA; from removing the genera in question) and Z-scores are given for the 20 most important genera for classifying samples.

**OOB estimate of error rate: 42.37 %**				
Confusion matrix:	bench	kiosk	railing	class error
bench	39	13	7	33.9 %
kiosk	10	48	4	22.6 %
railing	19	22	15	73.2 %
**Most important genera in sample**				
Genera	bench	kiosk	railing	MDA
Prevotellaceae-Prevotella_9	0.005	0.004	0.001	0.003
Micrococcaceae-Arthrobacter	0.001	0.005	0.000	0.002
Moraxellaceae-Enhydrobacter	0.000	0.003	0.003	0.002
Lachnospiraceae-Agathobacter	0.003	0.000	0.002	0.002
Intrasporangiaceae-Ornithinimicrobium	0.004	0.001	0.000	0.002
Ruminococcaceae-Faecalibacterium	0.002	0.001	0.001	0.001
Lachnospiraceae-Coprococcus_3	0.002	0.002	0.000	0.001
Peptostreptococcaceae-Romboutsia	0.002	0.001	0.001	0.001
Rhodobacteraceae-Rubellimicrobium	0.001	0.002	0.001	0.001
Corynebacteriaceae-Corynebacterium_1	0.001	0.002	0.000	0.001
Burkholderiaceae-Variovorax	0.000	0.002	0.001	0.001
Microbacteriaceae-Rathayibacter	0.001	0.003	0.000	0.001
Geodermatophilaceae-Geodermatophilus	0.000	0.002	0.001	0.001
Micrococcaceae-Rothia	0.001	0.001	0.001	0.001
Actinomycetaceae-Actinomyces	0.001	0.002	0.001	0.001
Family_XI-Peptoniphilus	0.002	0.002	-0.001	0.001
Streptococcaceae-Anthococcus	0.000	0.002	0.000	0.001
Prevotellaceae-Prevotella_7	0.001	0.000	0.002	0.001
Rhodobacteraceae-Paracoccus	0.000	0.003	0.000	0.001
Bifidobacteriaceae-Gardnerella	0.001	0.001	0.001	0.001

Figure S1 – The significant predictors of qPCR 16S rRNA gene copy yields in air samples.

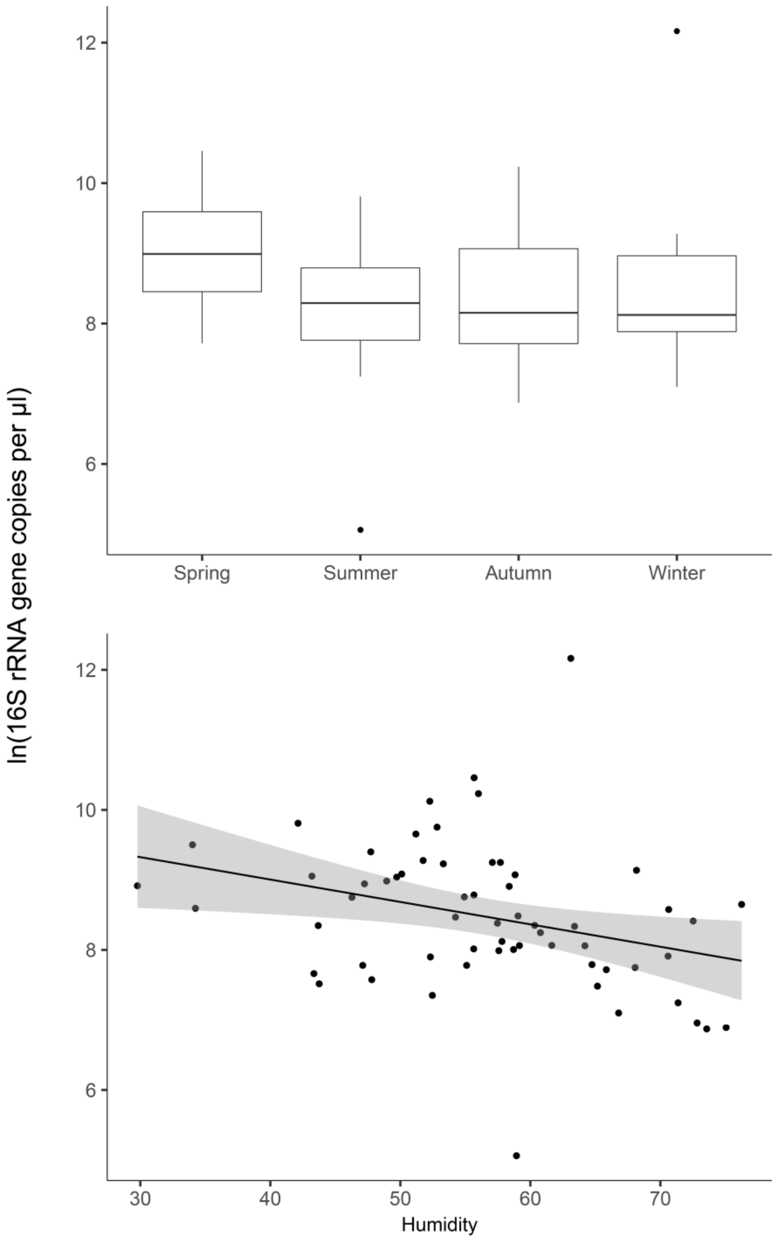

Figure S2 – The significant predictors of qPCR 16S rRNA gene copy yields in surface samples.

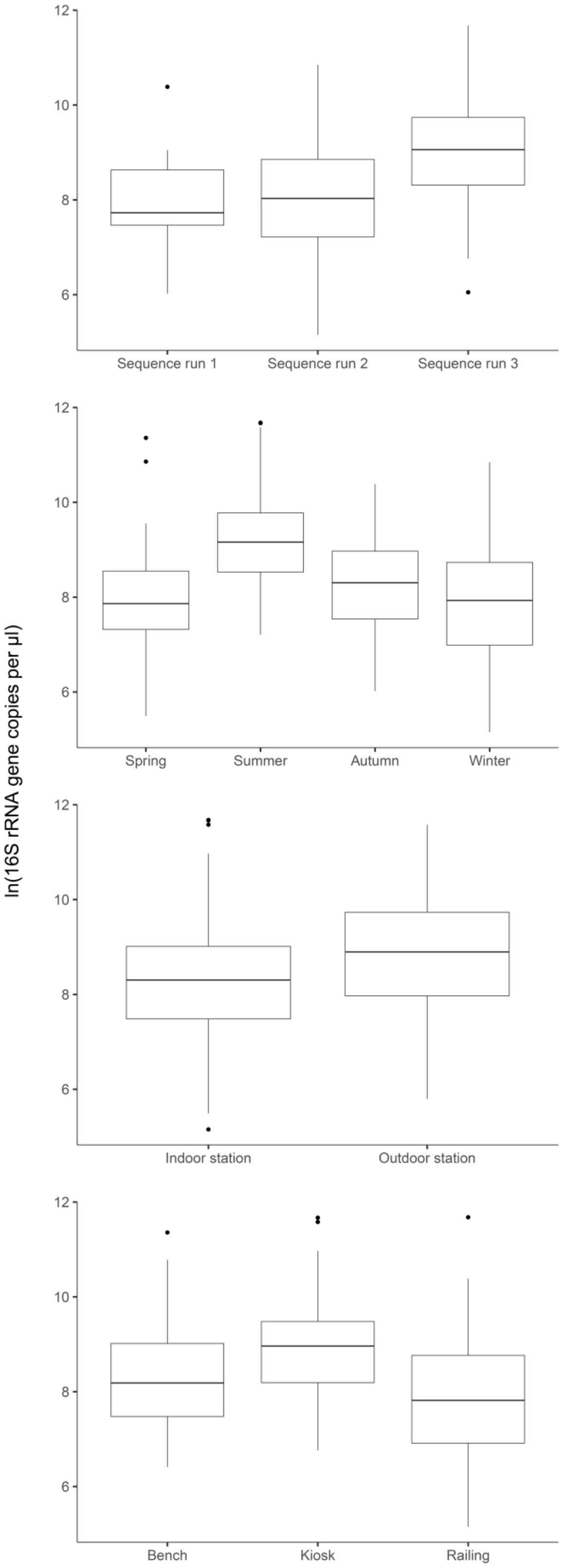

Figure S3 – Quality profile of filtered reads (forward reads only). The quality score (QS) distribution is shown as a grey-scale heat map, where dark colors indicating higher frequency. The green is the mean QS, orange the median, and dashed orange lines give the 25th and 75th quantiles.

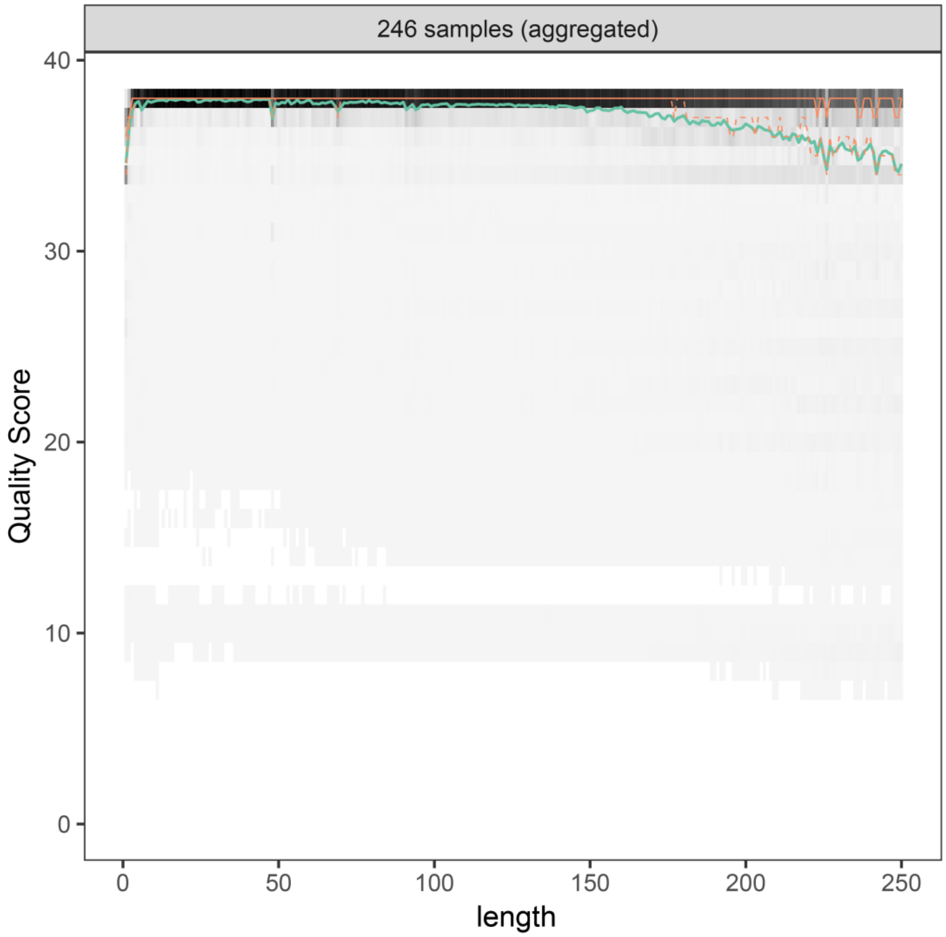

Figure S4 - Rarefaction curves with observed diversity and Shannon's Diversity Index. Diversity scores were obtained/calculated for sample sizes (reads) from 1 to 176394 (maximum sample size), with increasing interval size, using 5 iterations for each sample (1420 calculations * two alpha diversity metrics). Mean values from iterations are shown. Panel A shows rarefication curves to the rarefication level used (smallest sample size; 6358), and B shows rarefication plots for all available sample sizes.

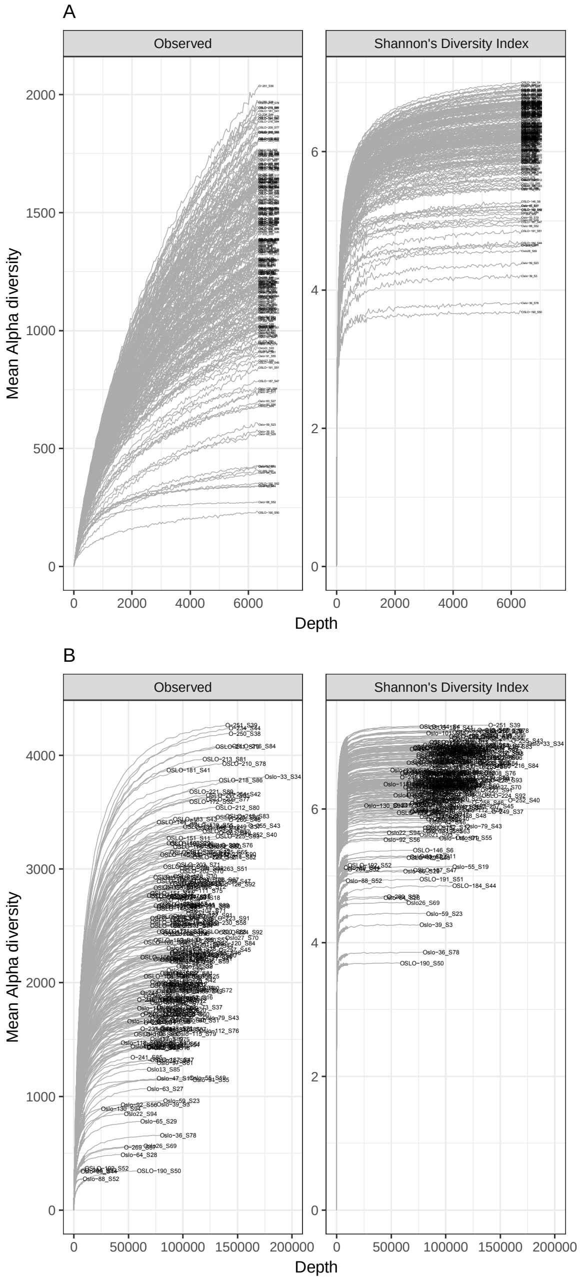

Figure S5 - A: Relative abundances of the top 15 phyla across the three surface types and seasons. **B**: Heatmap of most abundant families (relative abundance ≥ 0.01), color coded by phylum following the legend in panel A.

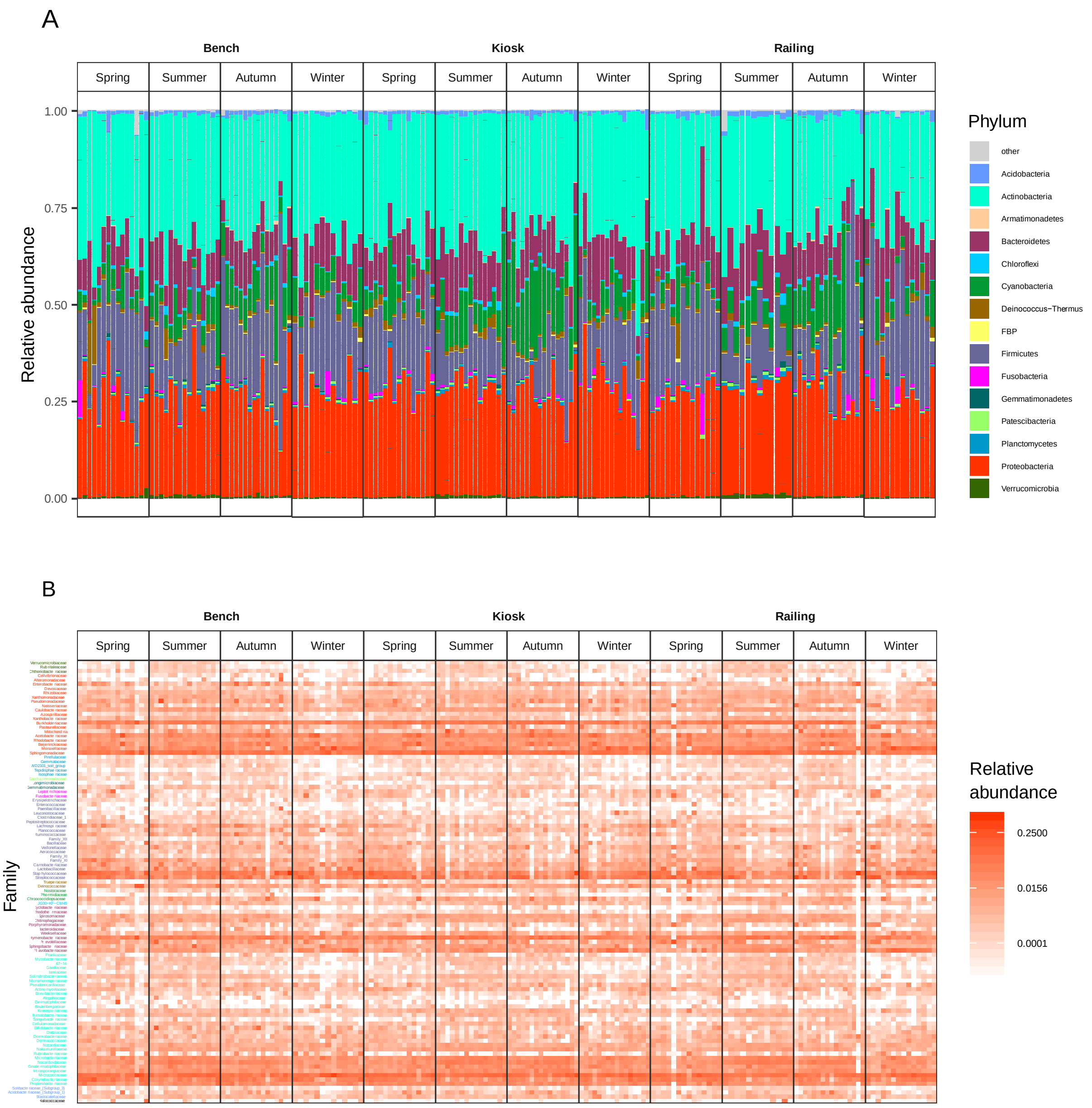

Figure S6 - Top 20 most important genera in random forest classification analysis of samples collected in different seasons.

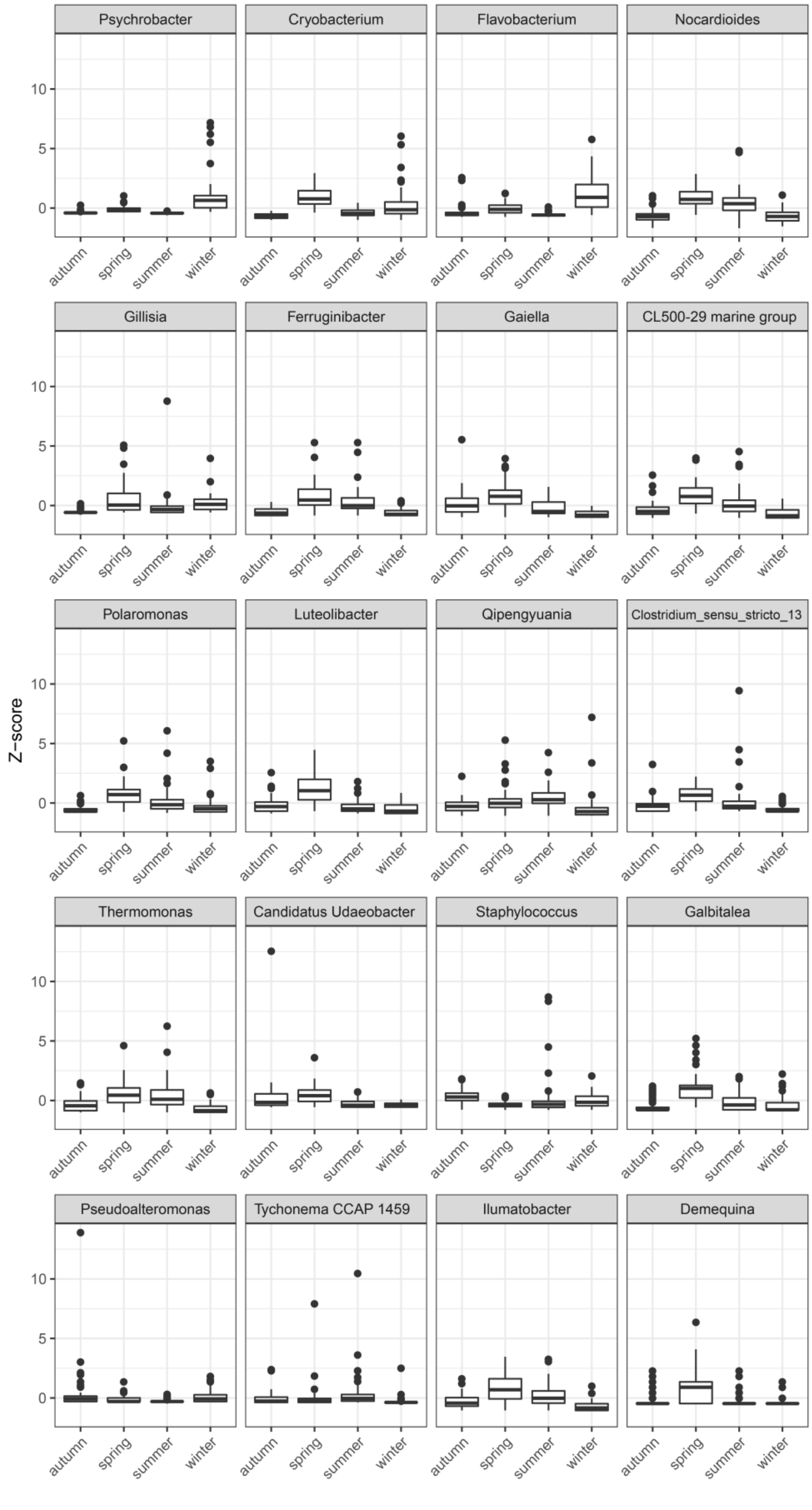

Figure S7 - Top 20 most important genera in random forest classification analysis of air and surface samples.

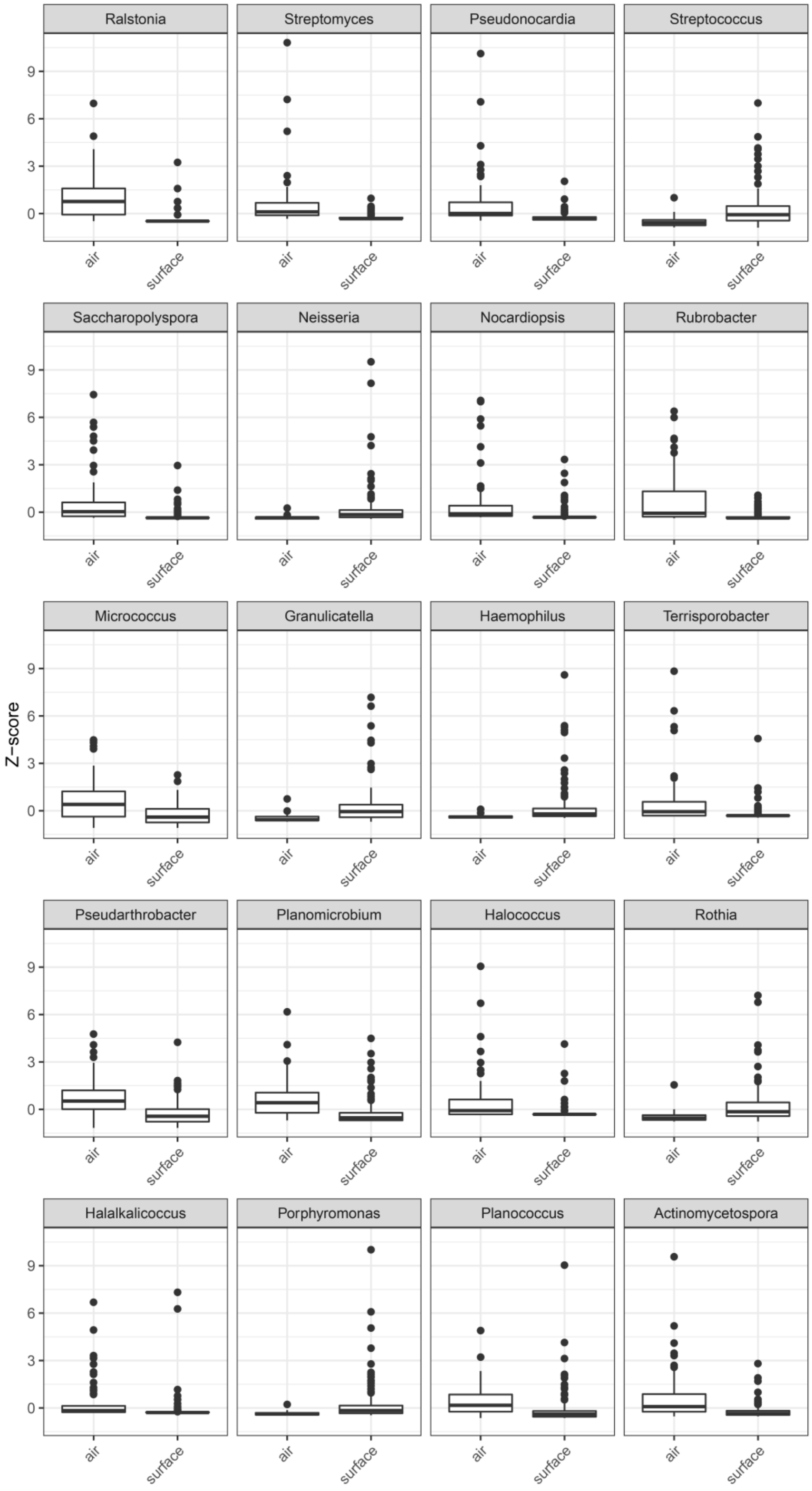

Figure S8 – Interaction effect between temperature (°C) and air/surface in the linear model of Shannon's diversity index. The reference level of air/surface is air; hence, the effect size refers to how much larger or smaller the Shannon's score is for surface samples, compared to air samples, at different temperatures.

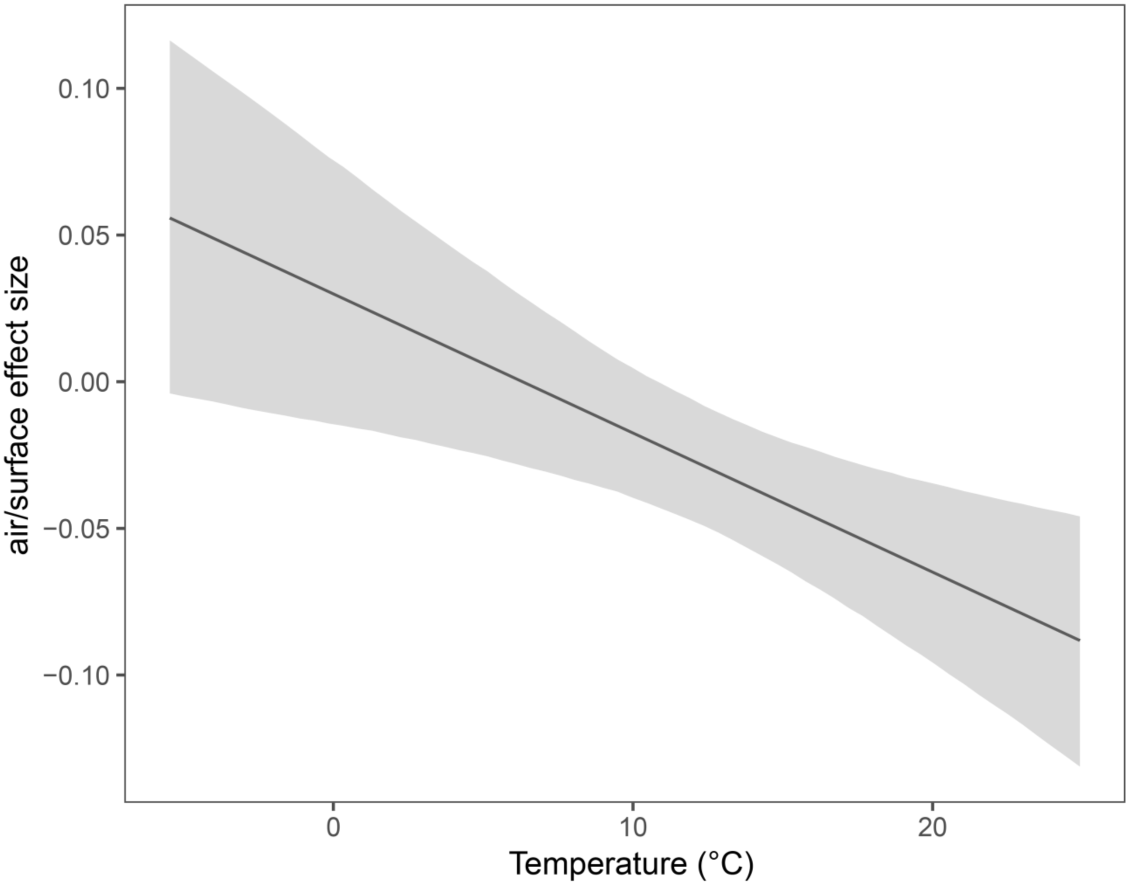

Figure S9 – PCoA plot of Bray Curtis dissimilarity distances with the only significant predictor (surface type) from the PERMANOVA model that included only surface-specific predictors (surface type, material and coating). Dashed circles represent 95 % CI for each cluster.

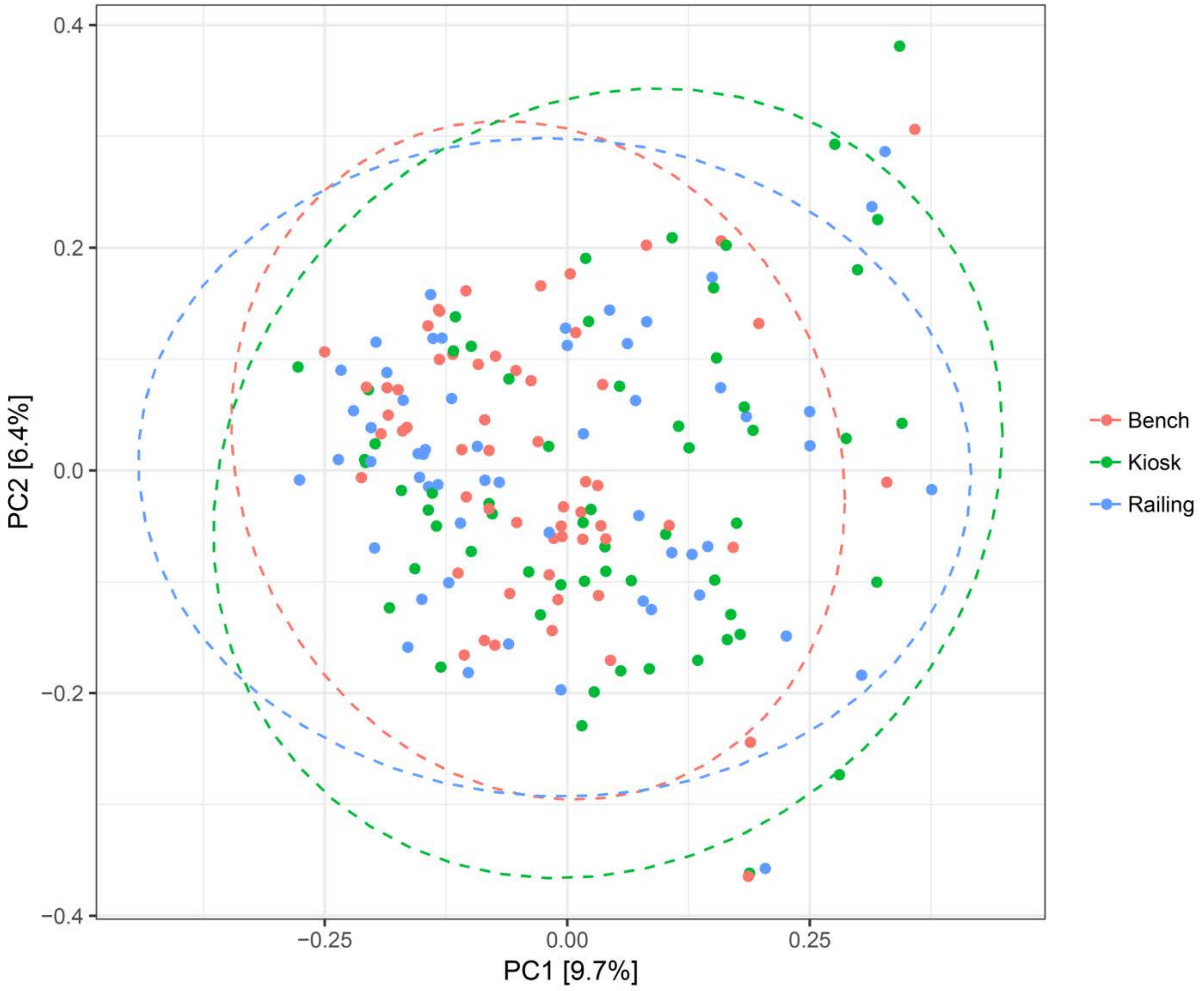

Supplement: Supplementary file 2 — Additional file 1: Table S1. Type of environment, latitude and longitude for all sampled stations. Table S2. Overview of all samples included in the analyses. Table S3. PCR program for 16S rRNA gene amplicon sequencing. Table S4. The best-fit models of qPCR 16S rRNA gene copies for air samples and surface samples. Table S5. Top 20 phyla, families, and genera and species in surface samples collected on kiosks, benches, and railings. Table S6. Random forest classification models of samples collected from different surface types. Figure S1. The significant predictors of qPCR 16S rRNA gene copy yields in air samples. Figure S2. The significant predictors of qPCR 16S rRNA gene copy yields in surface samples. Figure S3. Quality profile of filtered reads. Figure S4. Rarefaction curves with observed diversity and Shannon’s Diversity Index. Figure S5. A) Relative abundances of the top 15 phyla across the three surface types and seasons. B) Heatmap of most abundant families. Figure S6. Top 20 most important genera in random forest classification analysis of samples collected in different seasons. Figure S7. Top 20 most important genera in random forest classification analysis of air and surface samples. Figure S8. Interaction effect between temperature (°C) and air/surface in the linear model of Shannon’s diversity index. Figure S9. PCoA plot of Bray Curtis dissimilarity distances with the only significant predictor (surface type) from the PERMANOVA model that included only surface-specific predictors. [file 40168_2019_772_MOESM2_ESM.pdf]
